# Population density and water balance influence the global occurrence of hepatitis E epidemics

**DOI:** 10.1038/s41598-019-46475-3

**Published:** 2019-07-11

**Authors:** Anna Carratalà, Stéphane Joost

**Affiliations:** 1 Environmental Chemistry Laboratory (LCE), School of Architecture, Civil and Environmental Engineering (ENAC), École Polytechnique Fédérale de Lausanne (EPFL), Lausanne, Switzerland; 2 Laboratory of Geographic Information Systems (LASIG), School of Architecture, Civil and Environmental Engineering (ENAC), École Polytechnique Fédérale de Lausanne (EPFL), Lausanne, Switzerland

**Keywords:** Ecological modelling, Viral hepatitis

## Abstract

In developing countries, the waterborne transmission of hepatitis E virus (HEV), caused by HEV genotypes 1 (HEV-1) and 2 (HEV-2), leads to the onset of large recurrent outbreaks. HEV infections are of particular concern among pregnant women, due to very high mortality rates (up to 70%). Unfortunately, good understanding of the factors that trigger the occurrence of HEV epidemics is currently lacking; therefore, anticipating the onset of an outbreak is yet not possible. In order to map the geographical regions at higher risk of HEV epidemics and the conditions most favorable for the transmission of the virus, we compiled a dataset of HEV waterborne outbreaks and used it to obtain models of geographical suitability for HEV across the planet. The main three variables that best predict the geographical distribution of HEV outbreaks at global scale are population density, annual potential evapotranspiration and precipitation seasonality. At a regional scale, the temporal occurrence of HEV outbreaks in the Ganges watershed is negatively correlated with the discharge of the river (r = −0.77). Combined, our findings suggest that ultimately, population density and water balance are main parameters influencing the occurrence of HEV-1 and HEV-2 outbreaks. This study expands the current understanding of the combination of factors shaping the biogeography and seasonality of waterborne viral pathogens such as HEV-1 and HEV-2, and contributes to developing novel concepts for the prediction and control of human waterborne viruses in the near future.

## Introduction

Hepatitis E virus (HEV) is recognized as one of the most important agents of acute viral hepatitis worldwide^1^. According to estimates of the World Health Organization (WHO), every year there are around 20 million infections, 3 million of symptomatic cases of HEV and more than 50,000 deaths globally^1^. While in industrialized countries HEV infections normally lead to sporadic zoonotic or foodborne cases of acute hepatitis, in developing regions HEV causes large recurrent outbreaks affecting several hundred to several thousand persons^2^. Such outbreaks are caused by consumption or use of fecally contaminated water^3,4^. HEV waterborne outbreaks often occur in areas with limited access to clean water, or with low levels of water sanitation and hygiene. In addition, HEV waterborne outbreaks frequently strike areas of civil conflict or emergency such as refugee camps, where their control may be challenging and the disease outcomes more severe^5^.

HEV particles are small (approximately 27–34 nm in diameter), non-enveloped, and contain a single-stranded RNA (ssRNA) genome of 7.2 kilobases (kb). HEV is currently classified within the *Hepeviridae* family, and the *Orthohepevirus* genus and the *Orthohepevirus A* species^6^. Based on genetic analyses, human isolates of HEV are classified in four genotypes and several subtypes^6,7^. HEV genotypes 1 and 2 (HEV-1 and HEV-2, respectively) are responsible for the occurrence of large waterborne outbreaks in tropical and some subtropical regions^8^. Specifically, HEV-1 has been detected in Asian countries (Bangladesh, Cambodia, China, India, Kyrgyzstan, Myanmar, Nepal, Pakistan, Uzbekistan, and Vietnam) as well as in Africa (Algeria, the Central African Republic, Chad, Djibouti, Morocco, Sudan, Tunisia, Namibia, Egypt, and South Africa)^9,10^. Genotype 2 was initially detected in an epidemic in Mexico^11^, but more recently it has also been reported in African countries (Central African Republic, Chad, Democratic Republic of the Congo, Egypt, Namibia, and Nigeria^12–14^). Conversely, HEV genotypes 3 and 4 (HEV-3 and HEV-4, respectively) do not cause outbreaks but have instead been identified as causative agents in sporadic acute hepatitis E cases in the United States, Europe, China and Japan, and are only associated with cases of zoonotic origin^7^.

To date, HEV outbreaks due to HEV-1 and HEV-2, are thought to occur after events of heavy rainfall and floods, or in hot and dry months^2,15^. Rainfall and flooding may magnify the fecal contamination of water used for multiple purposes (drinking, cooking, personal hygiene, religious rituals, etc.). On the other hand, hot and dry months may possibly decrease the level of surface water leading to an increased concentration of enteric pathogens^16^. These observations suggest that climatic factors may be of major importance for the efficient transmission of HEV-1 and HEV-2 and the occurrence of outbreaks. However, the specific environmental conditions influencing HEV spatiotemporal dynamics remain largely unknown. It is therefore currently not possible to anticipate the spatiotemporal occurrence of HEV epidemics. In the context of climatic change, it is urgently needed to better understand the ecology of waterborne pathogens in order to increase our preparedness toward future outbreaks.

The analysis of health data, such as viral outbreaks or presence, from a geographical perspective (e.g. geographical distribution of the outbreaks) can highlight interesting patterns that can be used to formulate hypotheses about the role of different factors in the occurrence of an infection^17,18^. Species distribution models (SDMs), also known as niche models, are based on correlations between environmental data and geo-referenced presence/absence data (occurrence localities) of a particular species of interest^19,20^. These models are widely used in ecology and biogeography studies to address a broad array of questions, such as the identification of the potential distribution of an invasive species, mapping the risk of disease transmission, or anticipating the effects of climate change on the distribution of species of interest^21^. Among different distribution models, the maximum entropy method (MaxEnt) developed by Philips and colleagues a decade ago^22^ is one of the most widely used. This method exclusively relies on the use of presence data of a species of interest along with a set of environmental variables to model the species´ geographical distribution. This feature is particularly interesting to model the distribution of infectious diseases, such as HEV, for which absence data are rarely available. Presence data used in MaxEnt correspond to latitude-longitude pairs where a species has been observed, and environmental variables are layers of environmental information (e.g. temperature, amount of precipitation, a landcover class, etc.) in GIS format characterizing the same geographic area like the presence data records. Specifically, MaxEnt is based on a machine-learning algorithm which predicts environmental suitability for the species of interest given the environmental information, thereby estimating “a target probability distribution by finding the probability distribution of maximum entropy^22^”, thus containing a maximum of information. In other words, MaxEnt estimates what is closest to uniform distribution given a set of constraints based on current knowledge of the environmental variables at the occurrence sites^22^. Despite its popularity, the MaxEnt model does not directly estimate occurrence probability. Instead, it generates an index of relative habitat suitability. Other software, such as MaxLike, a more recent maximum-likelihood technique, has been shown to overcome the problem of directly estimating the probability of occurrence using presence-only data^23^. MaxEnt tends to under-estimate the probability of occurrence within areas of observed presences, but over-estimates it in unsampled areas beyond the spatial coverage of the data^24^. However, MaxEnt has shown to perform better than other SDMs^25–27^ and, unlike other software, it has been previously used in environmental virology to predict the distribution of multiple pathogens^28,29^.

In order to anticipate the spatiotemporal occurrence of HEV waterborne outbreaks at a global scale, we aim to: (i) identify spatial patterns in the occurrence of HEV epidemics, and (ii) determine the most important factors influencing such patterns. For this purpose, a database of publicly available data of HEV outbreaks was compiled, and this database was used in conjunction with environmental data to generate a global distribution model for the waterborne genotypes of HEV (HEV-1 and HEV-2). The results of this work expand our understanding of the factors promoting the spatiotemporal occurrence of HEV-1 and HEV-2, may contribute to anticipate the occurrence of waterborne outbreaks, and may ultimately be useful to optimize the control of waterborne pathogens in the next decades.

## Results

### Summary of the dataset and measures of risk

**Figure 1:**
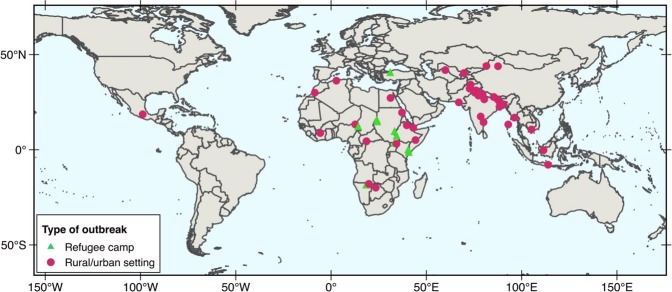
Outbreaks caused worldwide by hepatitis E virus (HEV-1 or HEV-2) due to the consumption or use of fecally-contaminated water included in the dataset compiled in this study. This map corresponds to outbreaks occurred from 1980–2017 affecting either population living in urban or rural settings, or in refugee camps.

The final dataset used in this study contains 59 geo-referenced outbreaks of waterborne HEV (caused by HEV-1 and HEV-2). To date HEV outbreaks have occurred in Africa, Asia and central America (Fig. 1). As shown in Table 1, most of the HEV outbreaks have been reported in Asia (59%) and in Africa (39%). More specifically, 27% of all reports of water-related outbreaks caused by HEV-1 and HEV-2 have occurred in the north of India (Ganges Valley). Notably, the main source of freshwater in the region, one of the most populated in the world is the Ganges river, which is the third largest river on the planet by discharge and highly polluted^30^. Another geographical region with recurrent occurrence of HEV outbreaks is equatorial Africa including Chad, Central African Republic, Sudan, South Sudan, Ethiopia, Somalia, Kenya and Uganda, among others. In these countries, the current number of recorded outbreaks also account for 27% of the total. Frequently, outbreaks in Africa occur in refugee camps^14,31^. Combined, the events recorded in the Ganges valley and the above-mentioned countries of Africa represent more than 53% of the total number of outbreaks reported to date globally. The rest of the outbreaks occurred in other Asian or African countries. Only one HEV outbreak (caused by HEV-2) has been reported outside these regions, namely in Mexico in 1986^32^.

**Table 1:** General summary of the dataset of hepatitis E waterborne outbreaks (1980–2017) compiled in this study, per continent.

Outbreak data	Europe	Africa	Asia	Australia	America
% of recorded outbreaks (n/total)	0 (0/59)	39 (23/59)	59 (35/59)	0 (0/59)	1.7 (1/59)
Average number of cases/outbreak	—	1,436	7,677	—	94
Cumulative incidence rate^a^	—	3.90	7.38	—	0.095
Incidence proportion^b^	—	0.006	0.015	—	0.0001

^a^Total number of cases (1980–2017) divided by the mean of the total population during the period x 100,000 population.

^b^Total number of cases (1980–2017) divided by the total population in 1980 × 100.

As shown in Table 1, the average number of cases per outbreak was higher in Asia than Africa (7,677 and 1,436 cases/outbreak, respectively). Similarly, the estimated cumulative incidence rate of HEV in Asia for the studied period (1980–2017) was 7.38 cases/100,000 population, whereas in Africa it was 3.90 cases/100,000 population. The incidence proportion was also higher in Asia than Africa (0.006 and 0.015%, respectively). The comparative risk (ratio of the incidence proportion) between Asia and Africa is thus 2.5, indicating that the risk of being infected by HEV in a waterborne outbreak is 2.5 times higher in Asia than in Africa.

### Models of spatial suitability for hepatitis E virus outbreaks

#### General model

**Figure 2:**
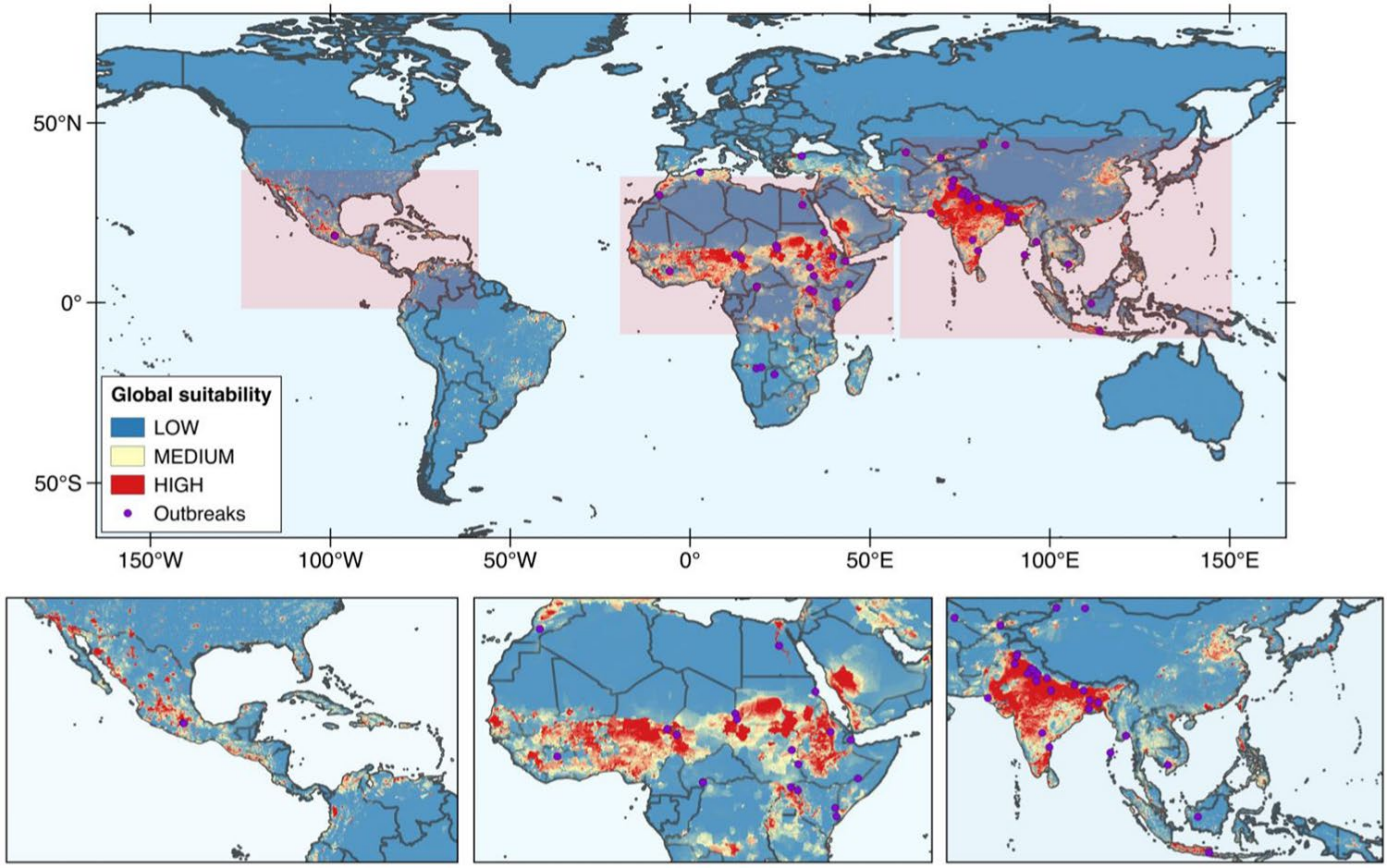
Map of the general model of the ecological suitability for the occurrence of hepatitis E waterborne outbreaks obtained by the Maxent method. This model was obtained combining outbreak data, population density and environmental data. Purple circles represent the location of the outbreaks used to obtain the model. The maps were obtained using obtained using the MaxEnt software version 3.4.1 (Steven J. Phillips, Miroslav Dudík, Robert E. Schapire. Maxent software for modeling species niches and distributions. Available from http://biodiversityinformatics.amnh.org/open_source/maxent/).

The combination of variables used in the general model (Fig. 2) resulted in an AUC value of 0.96 for the training data and 0.91 for the test data, while a value of 0.5 corresponds to a random prediction (Table 2). Plots showing how the model’s omission on test and training samples compare to the predicted omission, and its ROC curve and AUC values are shown in Figs S1(A) and S2(A) (supplementary information), respectively. The specific variables contributing the most to the general model (Table 2) were: the population density (by far the most important variable with a percent contribution and permutation importance of 80.9 and 76.3, respectively), the annual potential evapotranspiration (percent contribution and permutation importance of 11.4 and 12.2, respectively) and the precipitation seasonality (percent contribution and permutation importance of 3.8 and 1.4, respectively). Specifically, under this model the suitability for the occurrence of HEV increases with the population density, the annual potential evapotranspiration and the precipitation seasonality (Fig. S3, supplementary information). As seen in Fig. 2, when population density is included in the model, the geographical regions with highest ecological suitability for the occurrence of HEV outbreaks are India, Pakistan, Java Island and northeast China in Asia, the Pacific coast of Mexico, Colombia and Venezuela in central and South America, Morocco and other African countries above the Equator, and the region around Makkah (Saudi Arabia) and the Red Sea coast of Yemen in the Middle East.

**Table 2:** List of the variables used to develop the HEV distribution model based on environmental data and population density (global model) or with environmental data (environmental model).

Variable (id Worldclim dataset)	Global model (AUC = 0.91)		Environmental model (AUC = 0.90)	
	Percent contribution	Permutation importance	Percent contribution	Permutation importance
Population density	80.9	76.3	—	—
Annual potential evapotranspiration	11.4	12.2	56.9	7.1
Precipitation seasonality (bio15)	3.8	1.4	21.5	14.1
Mean diurnal range (bio2)	1.3	3.7	0.4	4
Precipitation of warmest quarter (bio 18)	1.1	1	1.6	2.4
Precipitation of driest quarter (bio 17)	0.7	1.5	8.4	2.6
Precipitation of coldest quarter (bio 19)	0.5	1.3	5.4	11.7
Mean temperature of wettest quarter (bio 8)	0.3	2.2	2.5	13.4
Soil topographic index	0	0	2.1	1.5
Mean temperature of driest quarter (bio 9)	0	0.2	1.3	43.2

The table also shows the percent contribution and importance in the final model for each variable. Variable id. corresponds to the identification of the variable in the Worldclim dataset. The percent contribution value depends on the algorithm path that MaxEnt used to obtain the model. The permutation importance depends exclusively on the final model obtained. AUC stands for “area under the curve” and PET stands for potential evapotranspiration.

#### Environmental model

**Figure 3:**
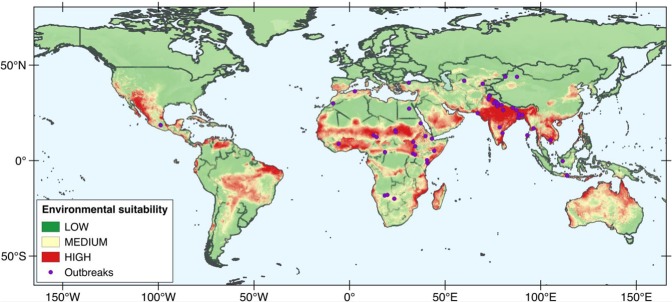
Map showing the environmental model of the ecological suitability for the occurrence of HEV outbreaks obtained using MaxEnt. To obtain this model, we combined only outbreak and environmental data. Population density data was excluded to highlight the effect of environmental parameters. The maps were obtained using obtained using the MaxEnt software version 3.4.1 (Steven J. Phillips, Miroslav Dudík, Robert E. Schapire. Maxent software for modeling species niches and distributions. Available from http://biodiversityinformatics.amnh.org/open_source/maxent/).

In order to highlight the role of environmental factors on the distribution of waterborne HEV outbreaks, we developed another model (named environmental model hereafter) using the same parameters used in the general model excepting the population density data. The AUC value of this model (shown in Fig. 3) was 0.89 for the training data and 0.90 for the test data. Plots showing how omission on test and training samples compare to the predicted omission, and the ROC curve and AUC values are shown in Figs S1(B) and S2(B) (supplementary information), respectively. The variables contributing the most to the model (Table 2) were the annual potential evapotranspiration (percent contribution and permutation importance of 56.9 and 7.1, respectively), the precipitation seasonality (percent contribution and permutation importance of 21.5 and 14.1, respectively) and, at a lesser extent, the precipitation of the driest quarter of the year (percent contribution and permutation importance of 8.4 and 2.6, respectively). In particular, under this model the predicted suitability for the occurrence of HEV outbreaks clearly increases with the annual potential evapotranspiration and the precipitation seasonality (Fig. S4, supplementary information). Generally, the model based on environmental information predicts a broader ecological suitability for the virus than the general model, which includes data of population density (Fig. 3). For example, the environmental model expects a high ecological suitability for the virus in the north of Australia and in Central and South America (especially, Brazil) while both locations are not particularly suitable if we take into account population density data. Notably, no HEV outbreaks have ever been recorded in these areas. Other areas of high suitability under the environmental model are the state of Arizona in the United States, the south of Spain and the south-east of Asia.

#### African and Asian models

In order to identify differences between the variables that best predict the occurrence for HEV in Africa and Asia, additional models were obtained using only outbreak data of Africa or Asia (African and Asian model, respectively). In turn, for each subset of the data we obtained a general model (including population density data) and an environmental model (which only includes environmental information and not population density). The general model obtained with outbreak data from Africa have AUC values of 0.91 for the test data and 0.96 for the training data, and the environmental model showed AUC values of 0.94 for the test data and 0.94 for the training data (Table S1, supplementary data). Under the African model based on environmental and population density data, the most important variables are annual potential evapotranspiration (percent contribution and permutation importance of 46.6 and 37, respectively), population density (percent contribution and permutation importance of 34.4 and 26.2, respectively), and precipitation during the warmest quarter of the year (percent contribution and permutation importance of 10.9 and 8.6, respectively). If we only use environmental data and outbreak data from Africa, the most important variables are annual potential evapotranspiration (percent contribution and permutation importance of 64.7 and 15.3, respectively), and precipitation during the warmest quarter (percent contribution and permutation importance of 12 and 15.9, respectively) and precipitation during the driest quarter of the year (percent contribution and permutation importance of 8.7 and 0, respectively). Both models predict similar distribution of ecological suitability for HEV as compared to the general model obtained using the complete outbreak dataset. Nevertheless, under the African models, the HEV suitability in the Ganges Valley is remarkably lower than expected under the full-dataset general model (Fig. S3, supplementary data). This is surprising considering that this region is the one with more reported outbreaks to date globally. Conversely, the general and the environmental model obtained with outbreak data in Asia have AUC values of 0.98 for the test data and 0.99 for the training data (general model), and 0.95 for the test data and 0.96 for the training data (environmental model), respectively (Table S2). While these AUC values are much higher than those obtained by the African models or the full-dataset models, the HEV ecological suitability predicted by the models obtained with outbreak data from Asia are not consistent with the full model (Fig. S4, supplementary data). Particularly, the Asian models, only predict a high ecological suitability for HEV in the south-east Asia. As shown in Table S2, under the Asian models the most important variables are population density for the general model (percent contribution and permutation importance of 96.5 and 98.3, respectively); and for the environmental model mean temperature of the wettest quarter (percent contribution and permutation importance of 40.9 and 25.1, respectively), annual potential evapotranspiration (percent contribution and permutation importance of 20.5 and 7.5, respectively) and precipitation seasonality (percent contribution and permutation importance of 16.3 and 50.3, respectively).

#### Factors influencing HEV seasonality in the Ganges watershed

We developed a correlation analysis using average monthly data of environmental and hydrological parameters for the Ganges watershed over the year in order to determine what factors may influence the temporal occurrence of HEV waterborne outbreaks (Table S3 and Table 3). Our results indicate that while the number of cases/outbreak is not correlated to any of the variables included in the analyses, the actual number of outbreaks is negatively correlated with the Ganges discharge (r = −0.77).

**Table 3:** Correlation matrix of the monthly values of HEV cases/outbreak and number of registered outbreaks in the Ganges watershed, and the monthly average values of precipitation, temperature, potential evapotranspiration and river discharge. PET stands for annual potential evapotranspiration.

	Cases	Outbreaks	Precipitation	Temperature	PET	Ganges discharge
Cases	/	0.09	−0.29	−0.37	−0.34	−0.32
Outbreaks	−0.03	/	−0.65	−0.50	−0.08	**−0.77**
Precipitation	−0.31	−0.65	/	0.68	0.33	**0.96**
Temperature	−0.34	−0.50	0.68	/	**0.87**	**0.79**
PET	−0.35	−0.08	0.33	**0.87**	/	0.42
Ganges discharge	−0.29	**−0.77**	**0.96**	**0.79**	0.42	/

## Discussion

In the present study we have combined outbreak and environmental data to model the ecological suitability for waterborne genotypes of HEV (HEV-1 and HEV-2) at global-scale, and determine the most important factors which favor the onset of HEV waterborne epidemics over time and space. Our dataset has highlighted geographical differences in the morbidity of the virus. Specifically, the cumulative incidence rate and the incidence proportion for HEV are much higher in Asia as compared to Africa. As a result, according to our dataset, the risk of being infected by HEV is 2.5 times higher in Asia than in Africa. Morbidity indices such as the cumulative incidence rate and the incidence proportion are a ratio of the cases of an infection or disease, and the population at risk at a certain time and region. Thus, differences in the comparative risk may be thus explained by a higher number of cases or a lower number of population at risk. Given that some of the most populated countries are found in Asia (e.g. China, India, Pakistan or Bangladesh), we can possibly rule out lower numbers of susceptible individuals in Asia as compared to Africa. It is thus possible that the conditions found in highly suitable areas of Asia are more favorable for the waterborne transmission of HEV than those typically found in highly suitable areas of Africa. This hypothesis deserves further attention in the future.

The general model obtained in the present study combines HEV-1 and HEV-2 outbreak data with population density data and other environmental variables selected on the basis of current knowledge on hepatitis E virus. This model predicts a high ecological suitability in regions where HEV waterborne outbreaks are recurrently identified (e.g. India, Pakistan and African countries above the Equator)^31,33–35^. However, we have not found any reports of HEV outbreaks occurred in Saudi Arabia and only one outbreak has been registered to date in Mexico, while both regions have high predicted suitability under our general model^32^. Nevertheless, in consistence with our results some studies have shown that HEV seroprevalence in adult population living in the cities of Makkah and Djeddah (Saudi Arabia) is very high (18 and 15%, respectively)^36,37^, indicating that at least sporadic or subclinical HEV cases may occur in the region. Other seroprevalence studies conducted in the country have shown lower seroprevalence values in areas of low suitability according to our general model. For example, one study showed that HEV seroprevalence among adult population living in Riyad was around 8%^38^. Most of these studies have observed higher seroprevalence among non-Saudis as compared to Saudi nationals. Johargy and colleagues found that the seroprevalence among Saudi nationals was 15% while it was to 23% among non-Saudis^36^. A large proportion of immigration entering Saudi Arabia comes from countries with very high suitability for the occurrence of hepatitis E outbreaks such as India, Indonesia, Pakistan or Bangladesh, for instance (https://www.iom.int/world-migration). In these countries the seroprevalence for HEV may be as high as 40%^39^, thus migration fluxes may contribute to the occurrence of hepatitis E viruses in Saudi Arabia, where some areas are highly suitable for the occurrence of HEV outbreaks. In the light of these results, ensuring water sanitation, water treatment and hygiene in these locations is highly recommended for the control of HEV. It is worth mentioning that few outbreaks have been recorded in areas where the suitability for HEV estimated by our general model is low to moderate. For example, to date three HEV outbreaks have been reported in the south of Africa (Namibia and Botswana) and four in Central Asia (China and Tajikistan). While this may highlight some deficiency in the model, it may also suggest that favorable conditions may eventually occur in regions with low-moderate susceptibility for HEV triggering the onset of a HEV waterborne outbreak.

According to the general model, the main variables influencing the ecological suitability for HEV are: (i) the population density, (ii) the annual potential evapotranspiration, and at a lesser extent (iii) the precipitation seasonality. This indicates that the global occurrence of HEV-1 and HEV-2 outbreaks is associated both with the presence of the virus’ host and hydrological factors, in agreement with the fact that HEV epidemics are primarily waterborne. These results are consistent with those obtained in previous studies showing that HEV waterborne outbreaks occurred during periods of drought or after extreme rainfall events^2,15,40^. Combined, these findings may indicate that the occurrence of outbreaks is influenced by the concentration of the virus in water. Among other factors, pathogen concentration in surface water and their transmission may be in turn influenced by either evaporation of polluted water, or by runoff after heavy rainfall events^16,41^. In regions with poor hygiene, low sanitation and where open defecation is commonly practiced, runoff after rainfall events can be particularly important as a source of human pathogens for rivers and lakes^42^.

Comparison between the general and the environmental model shows that if population density is not taken into account, the ecological suitability for HEV predicted by the resulting model is much broader. In fact, under the environmental model the suitability for HEV is remarkably high in countries where no waterborne outbreak of HEV has even been recorded (Australia and Brazil, for instance). This observation suggests that in absence of proper sanitation, if human population density increases in these areas, the geographical distribution of HEV epidemics could expand. Overall, considering the environmental model obtained in this work the variables that best predict the distribution of HEV are: (i) the annual potential evapotranspiration, (ii) the precipitation seasonality, and (iii) the precipitation of the driest quarter of the year. Once again, our results suggest that hydrological factors influencing river discharge may contribute to trigger the occurrence of an HEV outbreak caused by the waterborne genotypes of HEV (HEV-1 and HEV-2).

Models obtained using HEV outbreak data from Africa and Asia have showed interesting differences between the most important variables predicting HEV-1 and HEV-2 outbreaks. Human population density and annual potential evapotranspiration are important predictors of the occurrence of HEV-1 and HEV-2 epidemics in Africa and Asia. However, precipitation seasonality is a good predictor under Asian but not in the African models. This suggests that while at global scale HEV outbreaks seem to be explained by the combination of population density, potential evapotranspiration and precipitation seasonality, at local scale other variables may also influence the spatiotemporal occurrence of HEV waterborne cases. This finding may indicate that the environmental conditions favoring the occurrence of HEV epidemics caused by genotypes 1 and 2 in Asia may differ from those in Africa. Notably, these results could explain the difference in HEV risk between Asia and Africa observed from our outbreak dataset.

The correlation analysis developed using monthly data of HEV-1 and HEV-2 outbreaks, environmental and hydrological variables in the Ganges watershed suggests that the higher is the river discharge, the fewer outbreaks occur. Noteworthy, our dataset suggests that the safer months in the region in terms of occurrence of HEV outbreaks are the monsoon months; June, July, August and September. Consistent with previous observations described above, our results highlight that ultimately water balance, and thus pathogen concentration in a watershed may influence the temporal occurrence of waterborne outbreaks caused by HEV. Some previous studies have shown that streamflow is correlated with biological contamination and that pathogen load is influenced by river discharge^16,43^. Our results are in agreement with a previous study investigating the presence of HEV RNA in sewage of northern India from 2004 to 2006^15^. In this study, the authors observed the presence of HEV in 46% of the tested samples in winter months (November to February), 55% in summer months (March to June) and 23% during the monsoon months (July to October). This study suggests that HEV load in the Ganges river may be indeed lower during the monsoon months. Conversely, one recent study has shown bimodal peaks for HEV cases in India, with a peak occurring during the cold months of the year and a second peak in August, during the monsoon period^44^. It is possible that HEV cases occur during the monsoon months but that the environmental conditions during this period have a negative influence on the occurrence of large outbreaks. Another possible explanation for this disagreement is that the methods used in both studies have different accuracy to determine the occurrence of HEV cases or outbreaks. In addition, we cannot exclude the potential influence of sociological factors, such as pilgrimage events and religious celebrations which may sporadically increase the dissemination of the virus at a certain location and time of the year. Further studies are needed to fully understand the specific interplay of water balance, the concentration of enteric pathogens in surface water and the occurrence of infections among the population.

In conclusion, we have identified geographical areas of high general and environmental suitability for the occurrence of waterborne outbreaks of hepatitis E virus (genotypes 1 and 2). Specifically, our results indicate that, at a global scale, the most ecologically suitable hotspots for these viruses are the Ganges Valley and Pakistan, the west coast of Saudi Arabia and subequatorial African countries. In addition, we have determined the most important factors (population density and water balance) for the spatial occurrence of HEV waterborne outbreaks at global scale, and for the seasonal occurrence of outbreaks at regional scale using the Ganges watershed as a case study. Nevertheless, our results have also shown that local particularities may also influence the onset of an outbreak, and thus further studies are necessary to better understand the role of other factors such as rainfall, soil properties, pollution source, land use or river network ephemerality acting at regional scale.

The dataset of HEV-1 and HEV-2 outbreaks used here is relatively small (59 entries) and the existence of geographical and temporal irregularities on the reporting and identification of HEV epidemics cannot be excluded. For example, changes in the sanitation level or population density in a certain region over the study period could certainly influence the occurrence of waterborne outbreaks and therefore change the general model obtained here. Similarly, it is a well-known fact that many waterborne diseases such as HEV are underreported due to the generally mild associated symptoms^45^. Due to the lack of spatially continuous data, in our models we have not included other environmental factors that may also be important for the occurrence of HEV outbreaks (e.g. sanitation). In addition, the environmental variables included in as covariate in the analyses were selected given current knowledge on the environmental transmission of the virus, but it is also possible that covariates are missing, representing a further source of modelling error. Similarly, we have not taken into account the potential role of cross-immunity in the population due to co-infection with HEV-3 and HEV-4 circulating in the territory. Some authors have shown that Rhesus monkeys infected with genotype 1 or genotype 4 developed immunity to infections caused by the other genotype, suggesting the possibility of cross-protection between waterborne and foodborne HEV genotypes^46^. Therefore, the circulation of HEV-1 and HEV-2 in regions with low sanitation conditions could influence the circulation and distribution of HEV-3 and HEV-4, and *vice versa*.

A vaccine against HEV is commercially available in China since 2011 (Hecolin®, Xiamen Innovax Biotech Co., Ltd., China) and recommended for individuals at high risk of HEV infections. To date, it has not been licensed in any other country and no other experimental vaccine has progressed to clinical trials. We cannot comment on the possible effect of the vaccination of risk groups in China on the occurrence of HEV outbreaks in the country since currently little is known about the actual use of the Hecolin® vaccine^47^.

Despite its limitations, the present study represents a step forward for anticipating and preventing the occurrence of hepatitis E epidemics due to the contamination of drinking and recreational water with HEV genotypes 1 and 2. Our data will be useful to plan future prospective studies and may contribute to understand the movement of viruses between different geographical areas of diverse suitability levels. Data on the concentration of HEV in surface water, and the occurrence of HEV waterborne cases throughout the year should be obtained in order to establish the potential link between the HEV dose in the environment and the transmission of the virus among people.

## Materials and Methods

### Dataset compilation

The dataset used in this work was compiled using literature records of waterborne outbreaks of hepatitis E available in the PubMed database and in WHO reports^1^. In this work we use the term “waterborne HEV” to refer to outbreaks of hepatitis E caused by either genotype 1 and 2. The criteria to include literature entries reporting HEV outbreaks were that (i) the literature records provide the geographical coordinates of the location where the outbreak was detected, and (ii) the literature records provide clear evidence that the reported outbreak was related to the consumption or use of contaminated water, or alternatively that the causative agent was proven to be HEV genotype 1 or 2. All outbreaks included in the final dataset (n = 59) were geo-referenced using Google Earth (http://www.google.com/earth/). A map showing the location of all our dataset entries is shown in Fig. S1 and a brief summary is presented in Table 1. More information about each outbreak is presented in the accompanying website of this work (hevepidemics.com/index.html). In addition, we will share our database for non-profit purposes upon request.

### Estimation of risk indices

Cumulative incidence rate, and incidence proportion (also known as risk) were calculated for each continent in the studied period (1980–2017) with data of total cases included in our dataset, and total population data taken from the World Bank population data (https://data.worldbank.org/) (Table 1). The cumulative incidence rate corresponds to the number of cases registered between 1980 and 2017 divided by the average population in the same period and multiplied by 100,000. Incidence proportion was calculated as the number of cases divided by the total population at the start of the studied period (1980).

### Environmental data

Nineteen layers of environmental data were downloaded from the WorldClim dataset (http://worldclim.org) with a spatial resolution of 2.5 arc-min. The bioclimatic variables in this dataset are derived from monthly temperature and rainfall values. Specifically, the variables represent annual trends (such as mean annual temperature or the annual precipitation), seasonality (as the annual range in temperature and precipitation) and extreme environmental factors (such as the temperature of the warmest month of the year or the precipitation of the driest quarter of the year). The Worldclim bioclimatic values are generated by interpolation of average monthly data registered in many global meteorological stations from the year 1950 until the year 2000. In addition to the bioclimatic variables, we also included global data of soil wetness^48^, potential evapotranspiration (http://www.cgiar-csi.org/data) and human population density at 2.5 arc-min resolution. Human population density data were obtained from the Gridded Population of the World dataset for the year 2015 (http://sedac.ciesin.columbia.edu), as a “worst case scenario” assuming that a higher population density is beneficial for the onset of waterborne outbreaks of hepatitis E, and that population density has increased in many regions of the world during the last decades.

In order to identify and exclude highly correlated environmental variables prior to developing the SDM we calculated the variance inflation factor (VIF) for each variable, and excluded those which showed values higher than 6. VIF quantifies the level of multicollinearity in a least squares regression analysis^49^. Out of the 21 initial variables included in the correlation analyses, we retained those detailed in Table 3, and used them to obtain the distribution model of HEV.

### Species distribution models (SDMs)

The compiled dataset was used to build species distribution models for the waterborne HEV genotypes using the MaxEnt algorithm^22^. Prior to developing the model, 25% of the dataset was randomly set aside as “test samples” in order to obtain the distribution model, which was simultaneously validated using the rest of the dataset records, “training samples” (representing the remaining 75% of the total number of outbreaks). To obtain more information on the factors influencing the spatial occurrence of HEV outbreaks we processed four different models: (i) a model using population density data, environmental data and our complete dataset of hepatitis E outbreaks (general model), (ii) a model using environmental data and our complete dataset of hepatitis E outbreaks (environmental model), (iii) models using population density data, environmental data and outbreaks included in our dataset registered in Africa or Asia (African or Asian general model, respectively), and (iv) models using environmental data and outbreaks of our dataset registered in Africa or Asia (African or Asian environmental model, respectively). The models for Africa and Asia were developed to identify differences on the conditions favoring the occurrence of outbreaks in each continent. For our MaxEnt runs we used version 3.3.3 k of the software with its parameters set as follows: the convergence threshold was 0.00001, a maximum of 10,000 background points and 1000 iterations, a regularization multiplier of 1, and a logistic output format^22^. We conducted cross-validation runs in order to validate our model using 10 independent runs with different training (considering a random test percentage of 25%) and testing datasets. This allows the program to generate some statistical analyses which are detailed below.

The environmental variables that best predict the spatial distribution of HEV outbreaks were determined by their percentage contribution (PC) and the permutation importance (PI) as calculated by MaxEnt. In addition, the software also provides a jackknife test for the training and testing gain, and a plot showing the receiver operating curve (ROC) and the area under the ROC curve (AUC). Once a model was built using the complete list of uncorrelated environmental variables, we developed additional models selecting exclusively the 5 most-important ones given their PC, PI and jackknife results. The subsets of variables leading to the model with highest AUC scores were the selected for the final model. The model outputs were visualized using QGIS v2.18 (https://www.qgis.org/). A web version of the maps presented in this study is available for exploration in the accompanying website: hevepidemics.com/index.html.

### Seasonal correlation analysis at a regional scale

**Figure 4:**
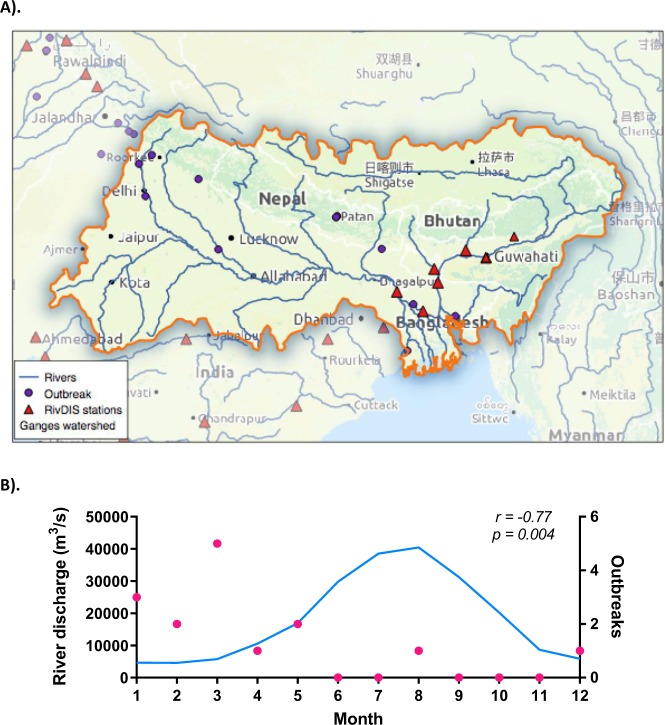
(**A**) Map of the Ganges watershed with the RivDIS locations, the registered outbreaks included in the dataset and the river network. Maps^©^ www.thunderforest.com, Data^©^ www.osm.org/copyright. (**B**) The blue line represents the monthly average values of the flow of the Ganges River (m^3^/s) registered in RivDIS stations. Red dots stand for the number of outbreaks recorded from 1980 until 2017 in the watershed area. The variables are negatively correlated (r = −0.77, p = 0.004).

To further investigate the environmental factors influencing the seasonal occurrence of HEV outbreaks at a regional scale, we used the Ganges watershed as a case study. The Ganges watershed (Fig. 4) is located within the Ganges-Brahmaputra basin, draining 1,086,000 km^2^ in Tibet, Nepal, India and Bangladesh and borders the Indus basin. The Himalayas are located to the north of the watershed. The Ganges catchment comprises the fertile Ganges plain and lies in the states of Uttar Pradesh (294,364 km²), Madhya Pradesh (198,962 km²), Bihar (143,961 km²), Rajasthan (112,490 km²), West Bengal (71,485 km²), Haryana (34,341 km²), Himachal Pradesh (317 km²) and Delhi (1,484 km²), as well as Bangladesh, Nepal and Bhutan. Notably, the Ganges basin has a population of more than 500 million people, thus being the most populated river basin in the world. The climate in the region is mostly subtropical. The Ganges basin receives nearly 1,000 mm of precipitation annually, mostly during the monsoon season (July, August and September). The average maximum temperature across the basin is around 30 °C in summer and 20 °C in winter.

We first determined the starting month for each outbreak of our dataset recorded in the Ganges watershed area and calculated the number of outbreak per month. We extracted average monthly data of precipitation, temperature, and sunlight radiation for each outbreak location in the Ganges watershed from the Worldclim dataset at 2.5 arcmin resolution. We also extracted monthly data of potential evapotranspiration (http://www.cgiar-csi.org/data). A standard Pearson linear correlation analysis was performed using the number of outbreaks/month and the monthly averages of environmental data.

## Supplementary information

Supplementary information
