# Supplementary material for: Population density and water balance influence the global occurrence of hepatitis E epidemics
Source: Sci Rep. 2019 Jul 11;9:10042. doi: 10.1038/s41598-019-46475-3 (PMC6624372; doi:10.1038/s41598-019-46475-3)
Supplement: Supplementary file 1 — Supplementary information [file 41598_2019_46475_MOESM1_ESM.pdf]

Supplementary information

Population density and water balance influence the global occurrence of hepatitis E epidemics

Anna Carratalà[1*], Stéphane Joost[2]

[1]Environmental Chemistry Laboratory (LCE), School of Architecture, Civil and Environmental Engineering (ENAC), École Polytechnique Fédérale de Lausanne (EPFL), Lausanne, Switzerland. [2]Laboratory of Geographic Information Systems (LASIG), School of Architecture, Civil and Environmental Engineering (ENAC), École Polytechnique Fédérale de Lausanne (EPFL), Lausanne, Switzerland. [1] Corresponding author, email address: anna.carratala@epfl.ch (A. Carratalà).

Figure S1. Plot showing how omissions of training and test samples vary with the choice of cumulative threshold and how this compares with the predicted omission for the general model (black line) (A) and the environmental model (B).

A)

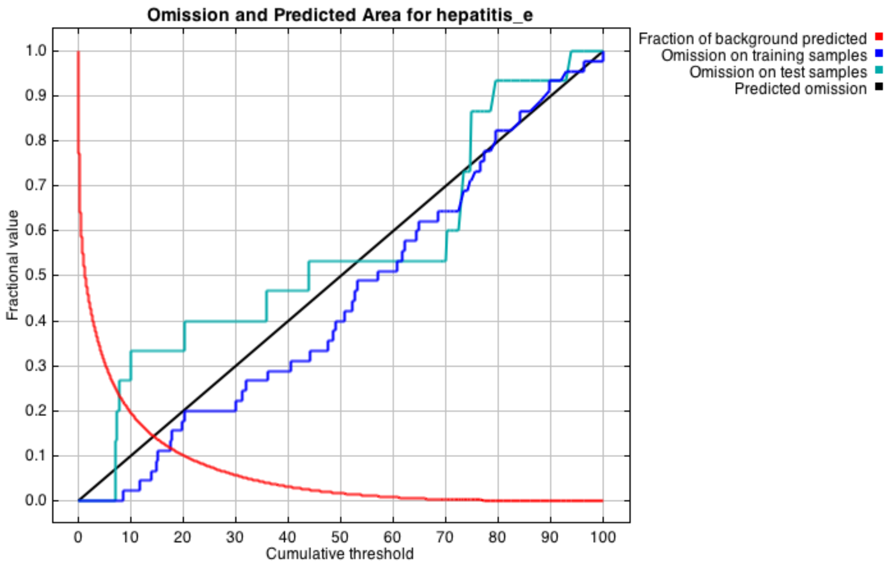

B)

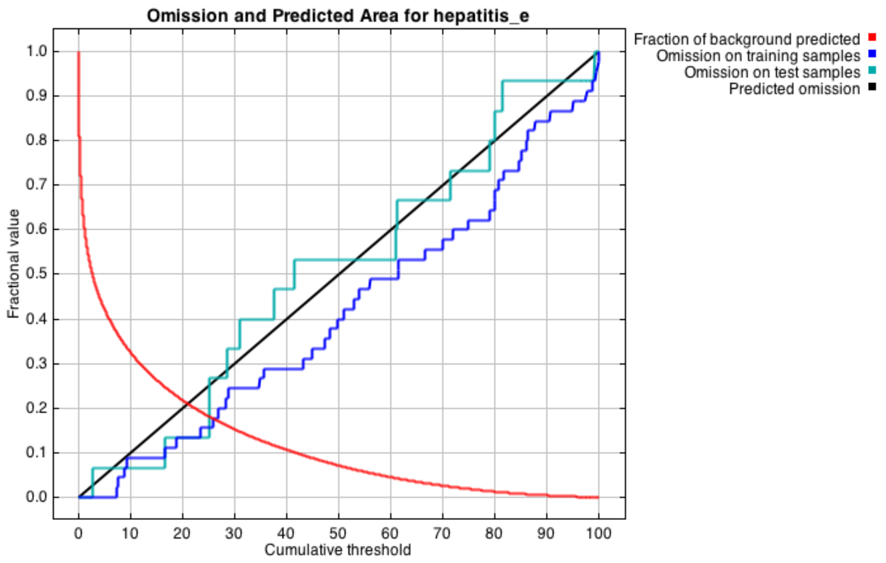

Figure S2. Plot showing the receiver operative curve for the training and test samples, as well as the AUC values for the general model (A) and the environmental model (B). An AUC value of 0.5 corresponds to a random prediction.

A)

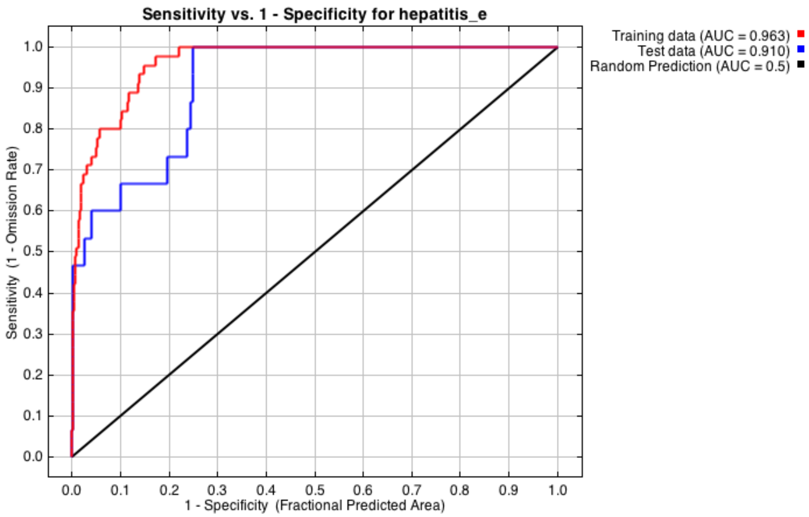

B)

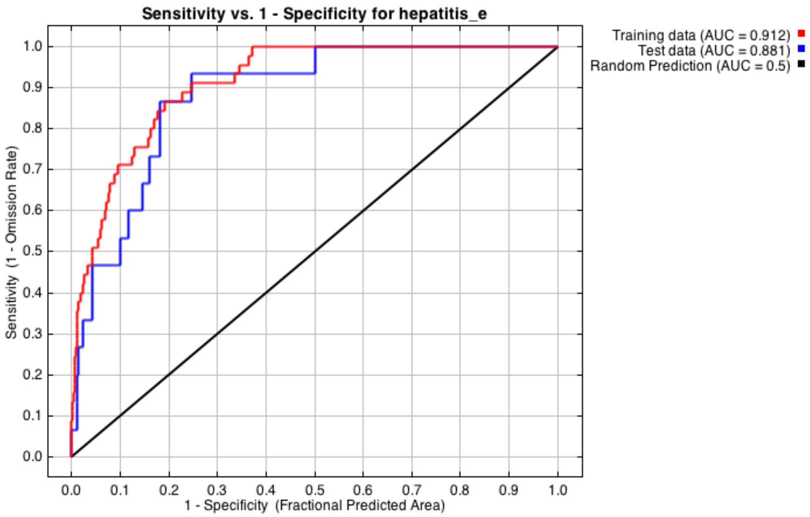

Figure S3. These curves show how environmental variables affect the Maxent model prediction under the conditions used for the general model. Each curve represents a different model created using only one variable at a time.

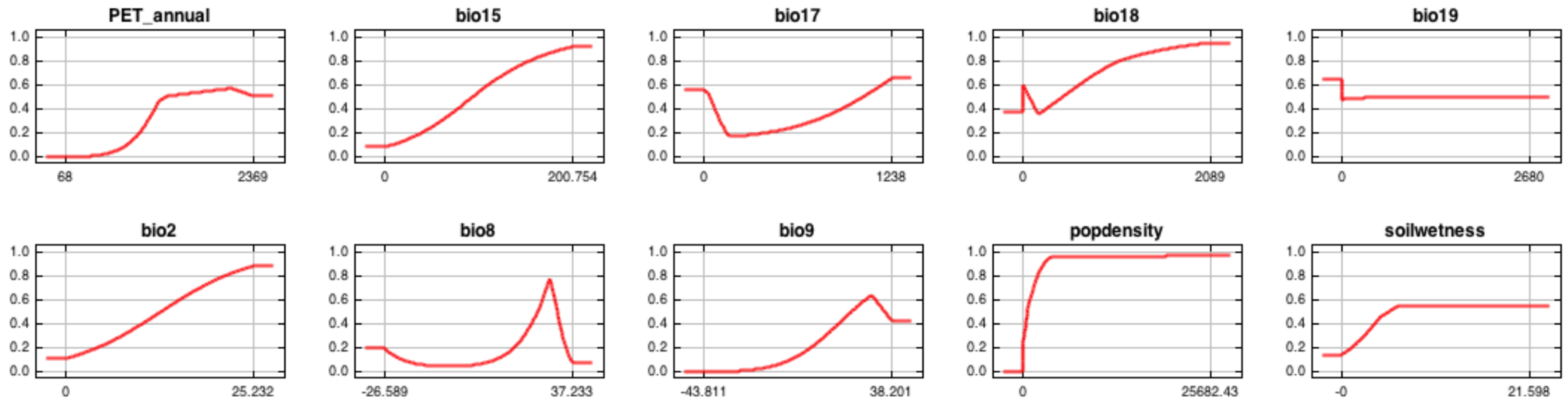

Figure S4. These curves show how environmental variables affect the Maxent model prediction under the conditions used for the environmental model. Each curve represents a different model created using only one variable at a time.

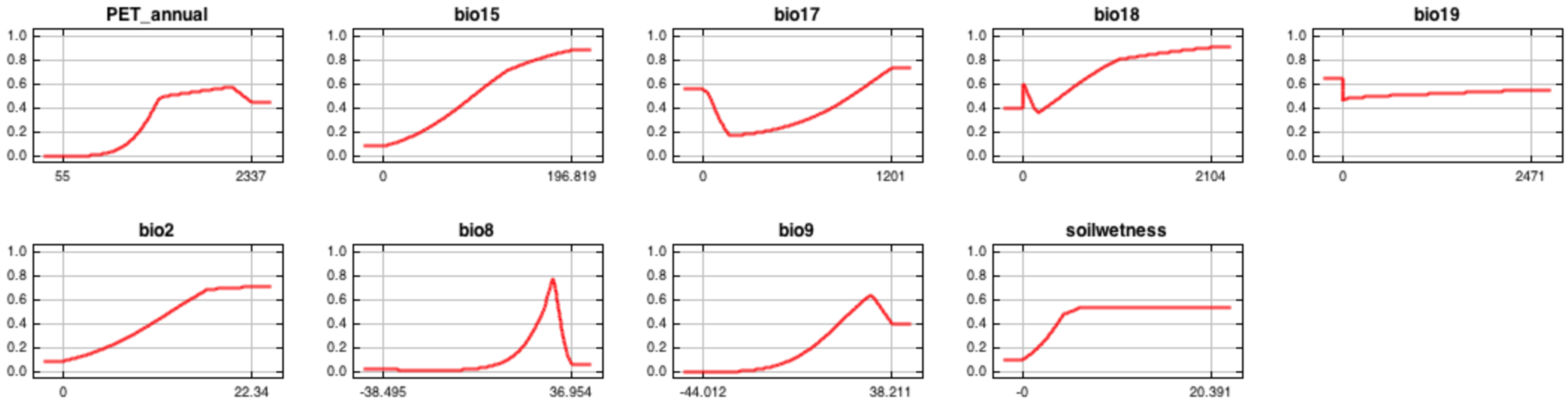

A).

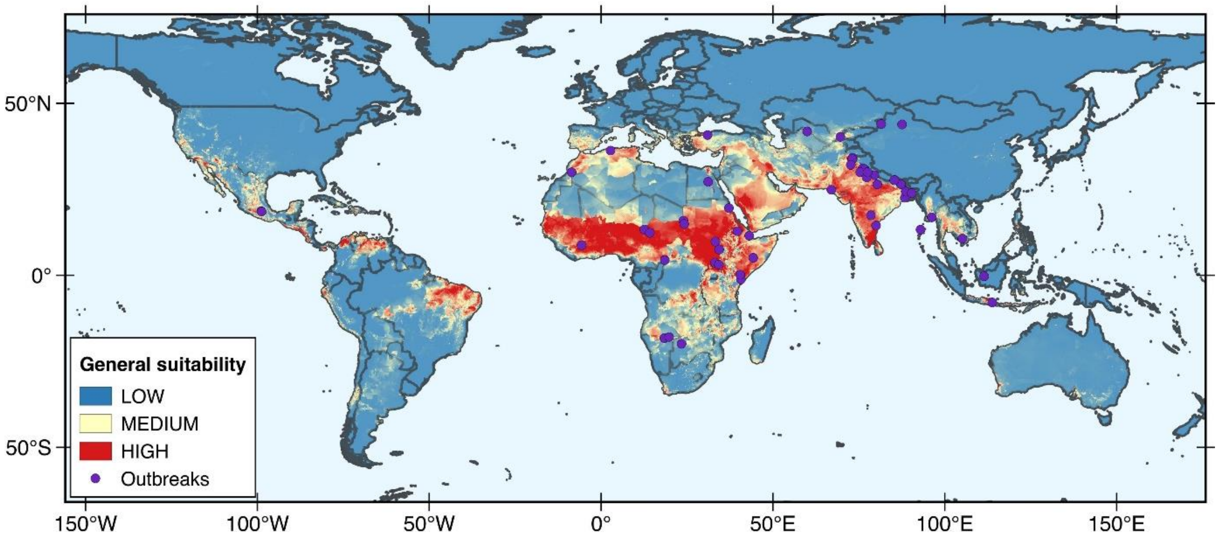

B).

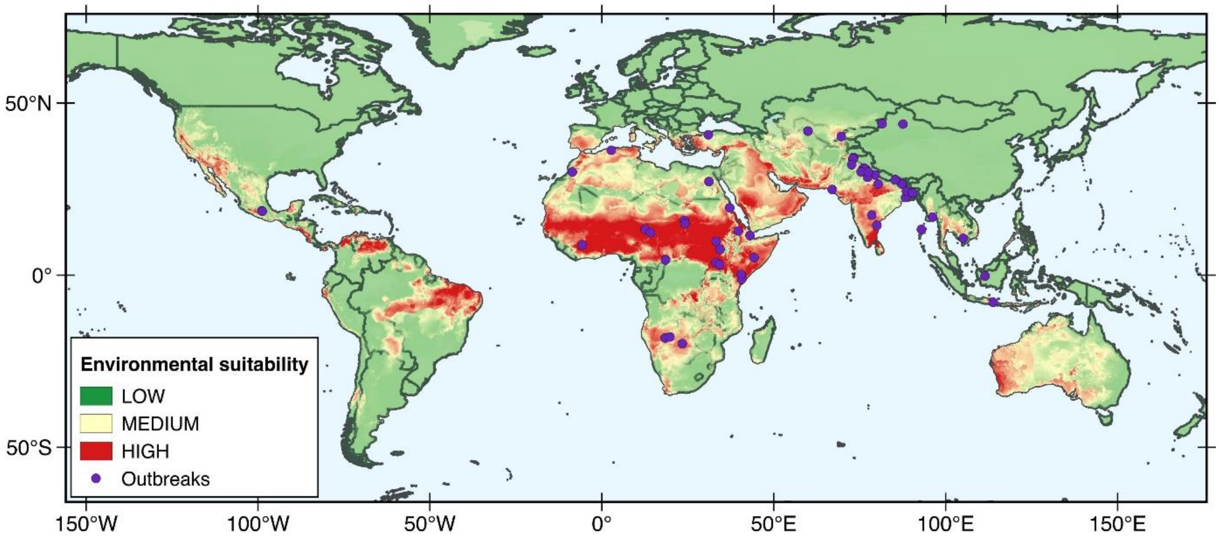

Figure S5. (A). Map showing the general model of the ecological suitability for the occurrence of HEV outbreaks. To obtain this model we combined outbreak data from Africa, population density and environmental data. (B). Map showing the environmental model of the ecological suitability for the occurrence of HEV outbreaks. To obtain this model we combined outbreak data from Africa and environmental data. Both maps also show the distribution of the entire HEV dataset compiled in this study, as a reference. The maps were obtained using obtained using the MaxEnt software version 3.4.1 (Steven J. Phillips, Miroslav Dudík, Robert E. Schapire. Maxent software for modeling species niches and distributions. Available from http://biodiversityinformatics.amnh.org/open_source/maxent/).

A).

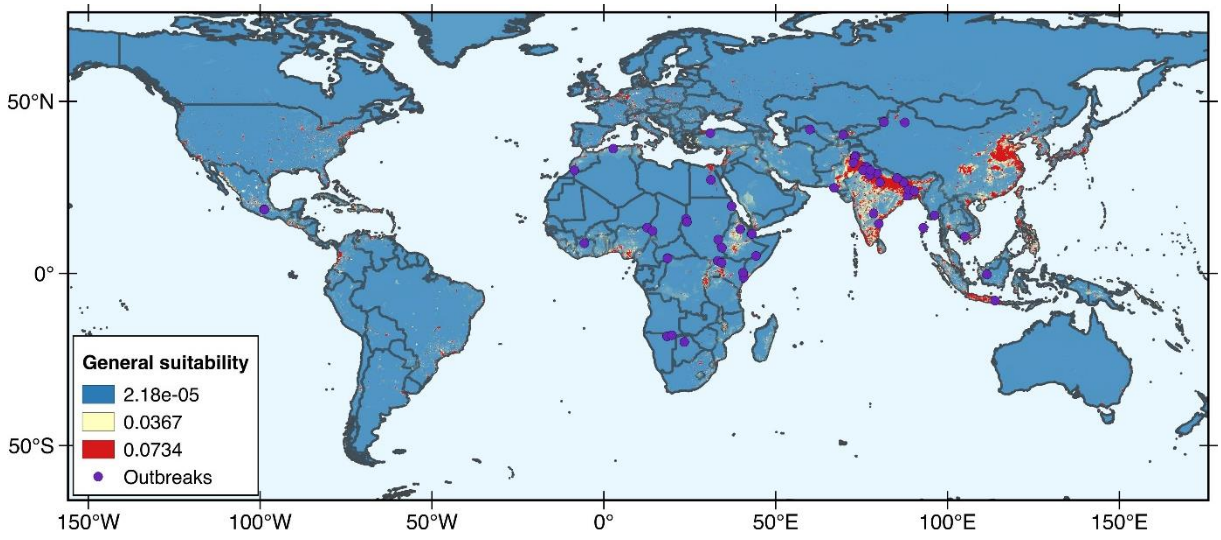

B).

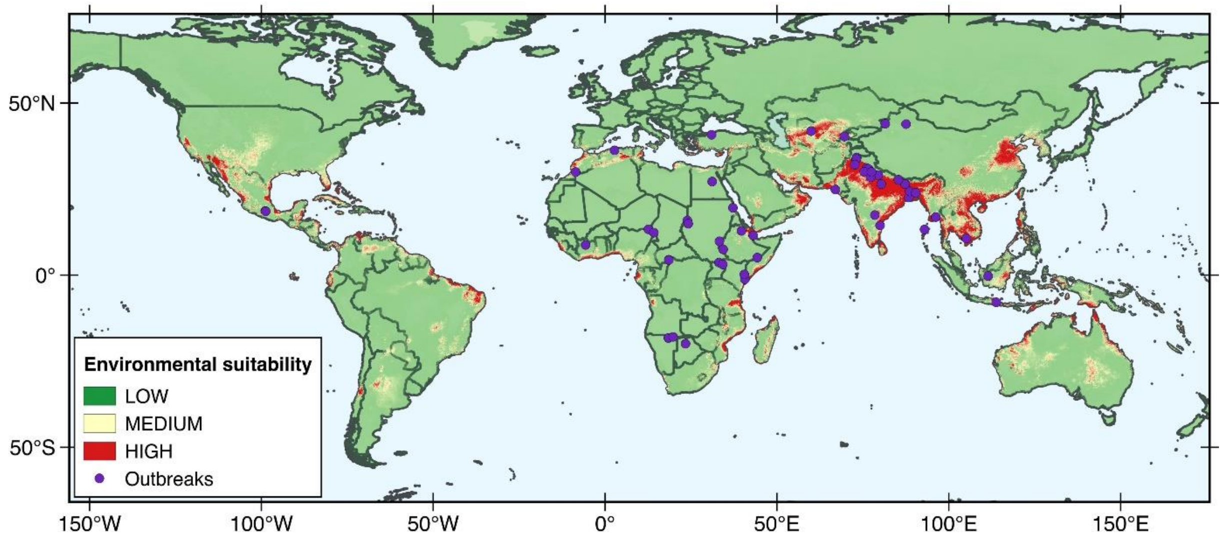

Figure S6. (A). Map showing the general model of the ecological suitability for the occurrence of HEV outbreaks obtained using MaxEnt. To obtain this model we combined outbreak data from Asia, population density and environmental data. (B). Map showing the environmental model of the ecological suitability for the occurrence of HEV outbreaks obtained using MaxEnt. To obtain this model we combined outbreak data from Asia and environmental data. Both maps also show the distribution of the entire HEV dataset compiled in this study, as a reference. The maps were obtained using obtained using the MaxEnt software version 3.4.1 (Steven J. Phillips, Miroslav Dudík, Robert E. Schapire. Maxent software for modeling species niches and distributions. Available from http://biodiversityinformatics.amnh.org/open_source/maxent/).

Table S1. List of the variables used to develop the HEV distribution model using the outbreaks occurred in Africa based on environmental data and population density (global model) or only with environmental data (environmental model). The table also shows the percent contribution and importance in the final model for each variable. Variable id. corresponds to the identification of the variable in the Worldclim dataset. The percent contribution value depends on the algorithm path that MaxEnt used to obtain the model. The permutation importance depends exclusively on the final model obtained. AUC stands for "area under the curve" and PET stands for potential evapotranspiration.

Variable (id Worldclim dataset)	Africa - Global model (AUC= 0.91)		Africa - Environmental model (AUC=0.94)	
	**Percent contribution**	**Permutation importance**	**Percent contribution**	**Permutation importance**
Population density	34.4	26.2	-	-
Annual PET	46.6	37	64.7	15.3
Precipitation seasonality (bio15)	0.1	0.4	0	0
Mean Diurnal Range (bio2)	0	0	0.1	1.1
Precipitation of Warmest Quarter (bio 18)	10.9	8.6	12	15.9
Precipitation of Driest Quarter (bio 17)	4.2	16.7	8.7	0
Precipitation of Coldest Quarter (bio 19)	2.9	1.9	6.3	3.9
Mean Temperature of Wettest Quarter (bio 8)	0.3	0.3	2.5	3.7
Soil topographic index	0	0	0	0
Mean temperature of driest quarter (bio 9)	0.7	8.8	5.6	60.2

Table S2. List of the variables used to develop the HEV distribution model using the outbreaks occurred in Asia based on environmental data and population density (global model) or only with environmental data (environmental model). The table also shows the percent contribution and importance in the final model for each variable. Variable id. corresponds to the identification of the variable in the Worldclim dataset. The percent contribution value depends on the algorithm path that MaxEnt used to obtain the model. The permutation importance depends exclusively on the final model obtained. AUC stands for “area under the curve” and PET stands for potential evapotranspiration.

**Variable (id Worldclim dataset)**	**Asia - Global model (AUC= 0.98)**		**Asia - Environmental model (AUC=0.95)**	
	**Percent contribution**	**Permutation importance**	**Percent contribution**	**Permutation importance**
Population density	96.5	98.3	-	-
Annual PET	0.5	0	40.9	25.1
Precipitation seasonality (bio15)	0	0	20.5	7.5
Mean Diurnal Range (bio2)	1.3	0.1	0.1	0.5
Precipitation of Warmest Quarter (bio 18)	0.1	0	0.6	1.9
Precipitation of Driest Quarter (bio 17)	0.4	0.5	7.7	4.6
Precipitation of Coldest Quarter (bio 19)	0.8	0.1	9.6	2.9
Mean Temperature of Wettest Quarter (bio 8)	0.4	0.9	16.3	50.3
Soil topographic index	0	0	3.5	0
Mean temperature of driest quarter (bio 9)	0.7	8.8	0.9	7.3

Table S3. Number of outbreaks occurred within the Ganges watershed included in our database according to their starting month. The total number of HEV cases (at the end of the epidemic period) and monthly average values of precipitation, temperature, potential evapotranspiration and river discharge in the Ganges watershed are shown.

Month	Nr. outbreaks	Cases	Precipitation (mm)	Temperature (°C)	PET (mm/month)	Ganges discharge (m^3/s)
January	3	385	25.94	14.04	77.75	4626.67
February	2	80364	20.69	16.67	92.50	4576.50
March	5	947	27.25	21.50	144.19	5768.87
April	1	104	31.63	26.36	179.31	10553.73
May	2	3848	73.69	29.36	202.00	16941.47
June	0	0	167.00	30.23	180.44	29811.00
July	0	0	330.88	28.56	149.69	38509.50
August	1	4751	299.38	28.03	136.81	40439.19
September	0	0	199.88	27.29	126.75	31213.53
October	0	0	70.19	24.43	121.69	20209.25
November	0	0	9.44	19.43	94.81	8678.94
December	1	3682	10.75	15.29	78.50	5865.83
